# Efficacy of photobiomodulation therapy using 980 nm versus 635 nm diode lasers for treatment of myofascial pain : a randomized controlled trial

**DOI:** 10.1186/s12903-025-06971-7

**Published:** 2025-10-02

**Authors:** Hala Shaaban Attiyah, Haytham Samir Moharrum, Usama Abd El Raouf M. El Dakrory

**Affiliations:** 1 National Institute of Laser Enhanced Sciences, Cairo University, Giza, Egypt; 2 Department of Oral and Maxillofacial Surgery, Sinai University, Al ‘Arīsh, Egypt

**Keywords:** Photobiomodulation, Myofacial pain, Low level laser therapy, TMJ myofascial pain, Surface electromyography

## Abstract

**Background:**

Myofascial pain syndrome (MPS) is a common musculoskeletal disorder characterized by myofascial trigger points and muscle dysfunction. Photobiomodulation therapy (PBMT) with diode lasers has shown promise for analgesia and functional improvement in MPS management.

**Aim:**

This study compared the efficacy of 635 nm and 980 nm diode lasers in alleviating pain and enhancing mandibular function in MPS patients.

**Methods:**

Thirty patients were randomized into two groups Group A (635 nm, 0.5 W, 60 s, 38.2 J/cm²) and Group B (980 nm, 0.2 W, 60 s, 15.3 J/cm²), applied twice weekly for five weeks. Outcomes included visual analogue scale (VAS), Maximum Mouth Opening (MMO), lateral/protrusive movements (LM/PM), and surface electromyography (sEMG) at baseline, post-treatment, and one-month follow-up.

**Results:**

Both groups showed significant improvements in all outcome measures (*p* < 0.001). VAS decreased from 7.6 ± 1.2 to 2.9 ± 1.3 (≈ 62% reduction) in the 635 nm group, and from 7.8 ± 1.1 to 2.1 ± 0.9 (≈ 73% reduction) in the 980 nm group (between-group *p* = 0.02). Maximum Mouth Opening (MMO) increased by 7.6 mm (34.5 ± 4.2 → 42.1 ± 3.2) in the 635 nm group versus 10.2 mm (34.0 ± 3.9 → 44.2 ± 2.9) in the 980 nm group (*p* = 0.01). Lateral movement (LM) improved by 2.3 mm vs. 2.8 mm, and Protrusive movement (PM) improved by 1.7 mm vs. 2.1 mm in the 635 nm and 980 nm groups, respectively. sEMG showed greater muscle activity reduction with 980 nm vs. 635 nm (Masseter: 47–51% vs. 39–40%; Temporalis: 43–44% vs. 36%; see(Table 3 for full data).

**Conclusion:**

PBMT with 980 nm produced superior pain relief, muscle relaxation, and Mandibular function recovery compared to 635 nm, likely due to deeper tissue penetration. However, interpretation should consider the small sample size and lack of placebo control.

**Trial registration:**

ClinicalTrials.gov, NCT07069764. Registered retrospectively on 07 July 2025.

**Supplementary Information:**

The online version contains supplementary material available at 10.1186/s12903-025-06971-7.

## Introduction

Myofascial Pain Syndrome (MPS) is a chronic musculoskeletal disorder marked by pain arising from myofascial trigger points (MTrPs) within taut muscle bands. These hypersensitive points can lead to both localized and referred pain, often manifesting as facial discomfort, headaches, shoulder pain, and mandibular dysfunction. MPS interferes with chewing, speaking, and yawning, often impairing sleep, psychological health, and quality of life [1, 2].

Travel in 1952^4^was the first to descTravell in 1952^4^ was the first to describe MPS and had been referred to by various terms, including myofascitis and fibromyositis. Clinicalribe MPS and had been referred to by various terms, including myofascitis and fibromyositis. Clinical manifestations of MPS often overlap with temporomandibular disorders (TMDs), with shared symptoms such as muscle and joint pain, TMJ sounds, limited or deviated mandibular movements, tinnitus, and dizziness. Etiological factors include trauma from dental procedures, muscular injuries, psychological stress, and anxiety [3–5].

Photobiomodulation therapy (PBMT), formerly known as low-level laser therapy (LLLT), is a non-invasive treatment increasingly used in dentistry. It employs low-intensity diode lasers (650–1000 nm) to stimulate cellular activity. PBMT enhances microcirculation, Adenosine Triphosphate(ATP) production, fibroblast activity, and lymphatic drainage. It also reduces inflammation by down regulating Cyclooxygenase-2 (COX-2) and Prostaglandin E2(PGE2)via photoreceptor activation. Though its exact analgesic mechanism is unclear, PBMT effectively reduces pain and inflammation. It also improves mandibular mobility and complements treatments like physiotherapy and occlusal splints [4–8]. 

Photobiomodulation therapy (PBMT) with diode lasers has shown potential for pain relief and functional improvement in myofascial pain syndrome (MPS). A recent scoping review in orthodontics demonstrated that low-level laser therapy (LLLT) can accelerate tooth movement and reduce pain, but highlighted substantial variability in protocols (Salha et al., 2025, BMC Oral Health). This emphasizes the need for a rigorously controlled and standardized design, as implemented in the present study, to reliably assess PBMT’s clinical effects [9]. 

Despite increasing use of PBMT for myofascial pain, there is a critical gap in the literature: few head-to-head clinical trials directly compare 635 nm and 980 nm diode lasers, leaving clinicians without clear evidence to guide wavelength selection in practice.

Mechanistically, 980 nm light penetrates deeper into muscle tissue, potentially reaching and modulating myofascial trigger points more effectively and influencing nociceptive pathways, whereas 635 nm primarily targets superficial tissues and microvascular responses.

This study was designed to address this gap by directly comparing both wavelengths under standardized parameters, assessing their effects on pain intensity, jaw function, and surface electromyography (sEMG) activity.

We hypothesized that 980 nm PBMT would yield greater long-term pain reduction, functional recovery, and muscle relaxation compared to 635 nm PBMT.

## Patients and methods

### Study design

This randomized controlled trial was conducted and reported in accordance with the CONSORT 2025 guideline.

A randomized controlled trial was designed to assess the effectiveness of low-level laser therapy (LLLT) in managing myofascial pain syndrome (MPS) associated with temporomandibular disorders (TMD). Thirty patients aged 18–60 years with clinically confirmed MPS were randomly allocated into two equal groups (*n* = 15): Group A received treatment with a 635 nm diode laser with flat top hand piece (Doctor Smile – Wiser 3 diode laser) (Italy), and Group B with a 980 nm diode laser with flat top hand piece(Doctor Smile – Wiser 2 diode laser)(Italy), both utilizing flat-top handpieces.

Both participants and outcome assessors were blinded to wavelength allocation to minimize performance and detection bias. Ethical approval was obtained from the National Institute of Laser Enhanced Sciences, Cairo University, and informed consent was secured from all participants.

### Sample size calculation

Sample size calculation was performed using Epi-Info v7.2.5.0, with 80% power, a 95% confidence interval, and α = 0.05, resulting in a final sample of 30 eligible patients. The primary outcome for the calculation was the difference in VAS pain scores between groups.

Based on previously published data and pilot observation, the expected difference between pre- and post-treatment VAS scores was approximately 2.0 units, with a standard deviation of 1.1. This effect size indicated that a minimum of 13 patients per group would be sufficient to detect a significant difference, but the sample was increased to 15 per group to account for potential dropouts.

Inclusion Criteria.

Participants were eligible if they met all of the following:

- Aged between 18 and 50 years.
- Diagnosed with stress-induced Myofascial Pain Syndrome (MPS).
- Medically healthy with no systemic diseases or conditions affecting muscle function.
- Not taking medications that could influence pain perception or muscle activity during the study period.
- Able and willing to comply with the study protocol and attend all treatment sessions.

Exclusion Criteria.

Participants were excluded if they had any of the following:

- Temporomandibular disorders (TMD) of bony or soft tissue origin.
- Presence of dental pain unrelated to MPS.
- Pregnancy or lactation.
- Chronic systemic illnesses (e.g., diabetes mellitus, cardiac arrhythmias).
- History of malignancy.
- Recent surgery involving the neck, shoulder, or jaw.
- Ongoing therapies that may affect muscle activity (e.g., physiotherapy, botulinum toxin injections).

### Intervention protocol

Laser therapy was administered twice weekly for five weeks (10 sessions). Before each session, laser output was verified with a calibrated power meter. A flat-top Handpiece ensured uniform energy distribution. Group A received 635 nm at 0.5 W for 60 s (fluence: 38.22 J/cm²), and Group B received 980 nm at 0.2 W for 60 s (fluence: 15.28 J/cm²). Both lasers operated in continuous wave (CW), with appropriate eye protection. Trigger points in masseter, temporalis, medial pterygoid muscles, and TMJ were palpated bilaterally, and laser was applied using standardized technique (1 cm² spot size). Parameters were selected based on previous clinical studies demonstrating efficacy in MPS and TMD; 980 nm targets deeper tissues, 635 nm affects superficial tissues, improving microcirculation and reducing inflammation (Monteiro et al., 2020; Awad et al., 2023; Al Quisi et al., 2023). Power and irradiation time were optimized for analgesic and functional outcomes while ensuring patient safety.

### Outcome measures

Patients were assessed at baseline, post-treatment, and 1 month post-treatment. Evaluation included:

Visual Analog Scale (VAS) for pain intensity, Maximum mouth opening (MMO), lateral and protrusive jaw movements measured with digital caliper, And Surface electromyography (sEMG) to assess masseter and anterior temporalis muscle activity before and after sessions. The examiner who recorded VAS and jaw movement outcomes was blinded to group allocation to minimize bias.

### Surface electromyography (sEMG)

sEMG was recorded using Neurosoft equipment (manufactured in Russia) to assess masseter and anterior temporalis muscle activity before and after sessions. Bipolar surface electrodes (~ 10 mm diameter, inter-electrode distance 2 cm) were placed bilaterally over the middle of the muscle belly, parallel to muscle fibers. Skin was prepared with alcohol and conductive gel to reduce impedance. Participants were seated upright with natural head posture and instructed to perform Maximum Voluntary Clenching (MVC) before and after treatment. Signals were amplified, filtered, and expressed in millivolts (mV) for statistical analysis of changes in muscle activity.


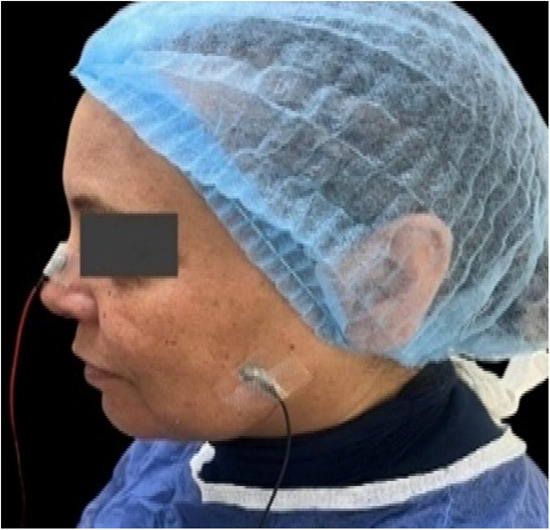


Image 1.electrode placement

### Statistical analysis

All data were non-normally distributed (Shapiro-Wilk test, *p* < 0.05); therefore, non-parametric tests were applied. Within-group changes were analyzed using the Friedman test, revealing significant improvements in all variables (VAS, MMO, LM, PM) in both groups (*p* < 0.001). Post-hoc comparisons with Bonferroni correction confirmed significant pre- to post-treatment and pre- to 1-month follow-up changes. Between-group differences were assessed with the Mann-Whitney U test, showing that the 980nm group achieved significantly greater improvements in all variables (*p* < 0.05). Effect sizes were large within groups (Kendall’s W = 0.68–0.79) and medium-to-large between groups (*r* ≈ 0.38–0.44), indicating clinically meaningful outcomes.

## Results

Of the final 30 participants in the study, 20 were females (66.7%) and 10 were males (33.3%) (Tables 1 and 2).

**Table 1:** VAS, MMO, lateral and protrusive movements in group A (635 nm, *n* = 15)

Variable	Pre-treatment Mean ± SD (Min–Max)	Post-treatment Mean ± SD (Min–Max)	1-month Follow-up Mean ± SD (Min–Max)	% Change (Pre→Post)	% Change (Pre→Follow-up)	*p*-value (Pre vs. Post)
VAS	7.6 ± 1.2 (5–9)	3.2 ± 1.1 (1–5)	2.9 ± 1.3 (1–5)	−57.9 %	−61.8 %	< 0.001
MMO	34.5 ± 4.2 (28–41)	41.3 ± 3.5 (36–47)	42.1 ± 3.2 (38–48)	19.7%	22.0%	< 0.001
LM	6.2 ± 0.9 (5–8)	8.3 ± 1.0 (7–10)	8.5 ± 0.8 (7–10)	33.9%	37.1%	< 0.001
PM	5.8 ± 0.7 (5–7)	7.4 ± 0.8 (6–9)	7.5 ± 0.7 (6–9)	27.6%	29.3%	< 0.001

**Table 2:** VAS, MMO, lateral and protrusive movements in group B (980 nm, *n* = 15)

Variable	Pre-treatment Mean ± SD (Min–Max)	Post-treatment Mean ± SD (Min–Max)	1-month Follow-up Mean ± SD (Min–Max)	% Change (Pre→Post)	% Change (Pre→Follow-up)	*p*-value (Pre vs. Post)
VAS	7.8 ± 1.1 (6–9)	2.5 ± 1.0 (1–4)	2.1 ± 0.9 (1–4)	−67.9 %	−73.1%	< 0.001
MMO	34.0 ± 3.9 (28–40)	43.5 ± 3.2 (39–48)	44.2 ± 2.9 (40–49)	27.9%	30.0%	< 0.001
LM	6.0 ± 0.8 (5–7)	8.7 ± 1.1 (7–10)	8.8 ± 1.0 (7–10)	45.0%	46.7%	< 0.001
PM	5.9 ± 0.6 (5–7)	7.8 ± 0.7 (7–9)	8.0 ± 0.6 (7–9)	32.2%	35.6%	< 0.001

Both groups showed improvement in pain and jaw function.

**Fig. 1:**
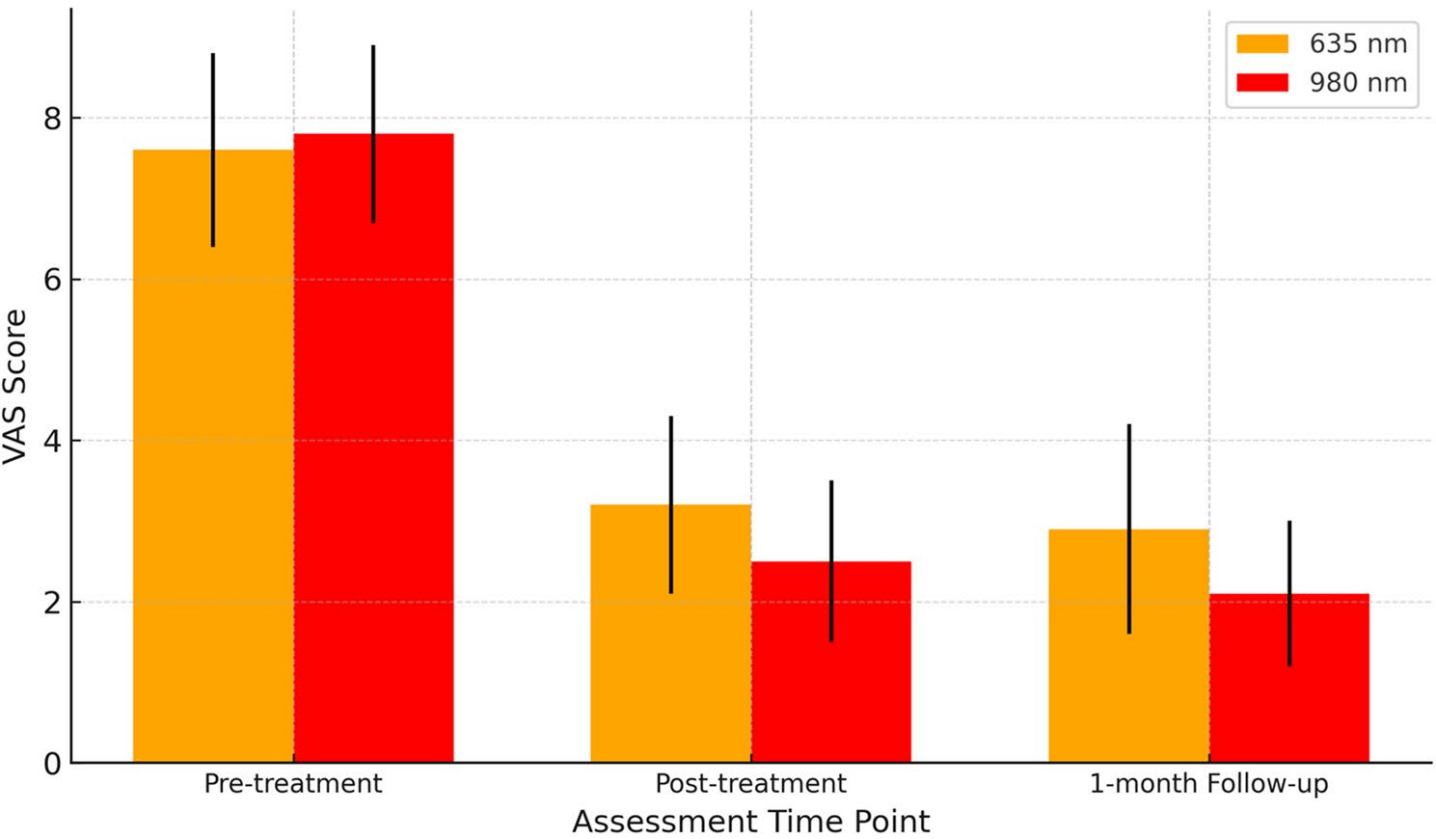
VAS scores over time (mean ± SD)
- Fig. 2: MMO improved more significantly in the 980 nm group than in the 635 nm group across all time points And one month follow up.

- Group 2 (980 nm) demonstrated greater mean improvements compared to Group 1 (635 nm), especially in VAS and MMO.
- MMO increased by approximately 8 mm in the 635 nm group and 10.1 mm in the 980 nm group.
- Improvements were Maintained at 1-month follow-up in both groups, indicating sustained therapeutic effects.
- Graphical insights

- Fig. 1: VAS scores (Mean ± SD) for the 635 nm and 980 nm groups at pre-treatment, post-treatment, and 1-month follow-up, showing greater pain reduction in the 980 nm group.

**Fig. 2:**
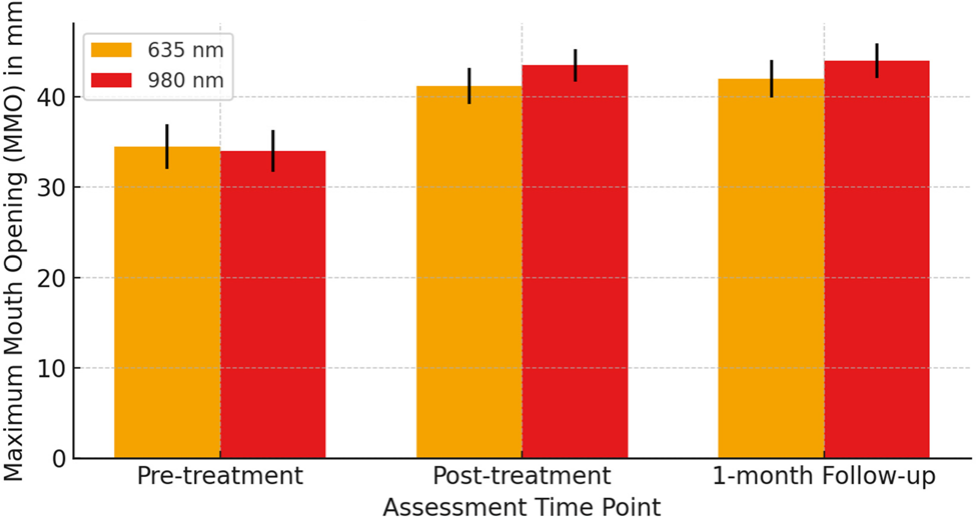
MMO improvements over time (mean ± SD)
- Fig. 3: Changes in lateral (LM) and protrusive (PM) Mandibular movements at pre-treatment, post-treatment, and 1-month follow-up for both groups. Group 2 (980 nm) showed superior improvements in both movement types compared to Group 1 (635 nm).

**Fig. 3:**
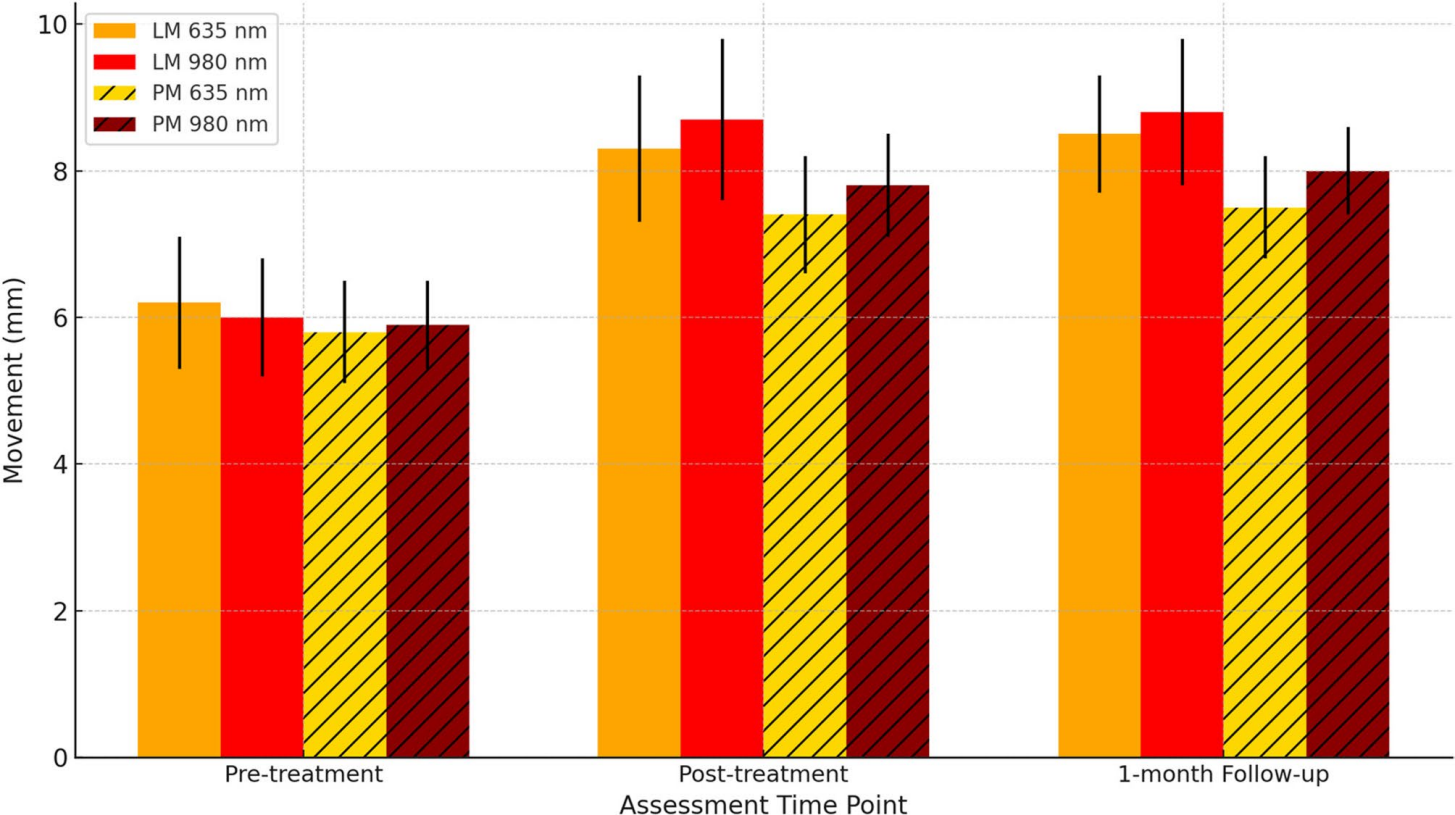
MMO improvements over time (mean ± SD)

Figures 1 to 3 show that the 980 nm diode laser is more effective than the 635 nm laser in reducing pain and improving jaw function in myofascial pain syndrome patients, with greater and longer-lasting benefits.

Surface electromyography (SEMG) results

- Both 635 nm and 980 nm diode laser therapies resulted in significant reductions in EMG activity across all muscles (*p* < 0.001, Friedman test).
- Post-treatment reductions were consistently greater in the 980 nm group (*p* < 0.05, Mann-Whitney U test).
- Both 635 nm and 980 nm diode lasers were effective in reducing myofascial pain and improving jaw mobility.
- 980 nm laser yielded superior outcomes in pain reduction, mouth opening, and muscle relaxation.

## Discussion

For treatment of myofascial pain, the first choice should be a conservative, and non-invasive method, such as PBM therapy.

The significant pain reduction observed in this study aligns with previous research supporting the effectiveness of PBMT in managing TMD-related myofascial pain. Fathy et al. (2021) [10] reported short-term benefits with 980 nm laser combined with splint therapy. Monteiro et al. (2020) [11] also demonstrated notable pain reduction using a 635 nm diode laser. Noor et al. (2024) [12] further confirmed these outcomes using 635 nm laser over four sessions, reporting significant improvement in pain intensity. Similarly, Farshidfar et al. (2023) [13] recent review highlighted PBMT’s role in improving pain and function, although the absence of standardized protocols remains a limitation (Table 3).

**Table 3:** sEMG activity of temporalis and masseter muscles in group A (635 nm, *n* = 15) and group B (980 nm, *n* = 15)

Muscle	635 nm Pre (Mean ± SD)	635 nm Post (Mean ± SD)	% Change (Pre→Post)	*p*-value	980 nm Pre (Mean ± SD)	980 nm Post (Mean ± SD)	% Change (Pre→Post)	*p*-value
Right Temporalis	15.5 ± 3.1	9.9 ± 2.9	−36.1%	< 0.001	15.8 ± 2.9	8.9 ± 2.3	−43.7%	< 0.001
Left Temporalis	15.9 ± 3.0	10.1 ± 2.8	−36.5%	< 0.001	16.1 ± 3.0	9.2 ± 2.4	−42.9%	< 0.001
Right Masseter	16.5 ± 3.1	9.9 ± 2.9	−40.0%	< 0.001	16.8 ± 2.9	8.9 ± 2.3	−47.0%	< 0.001
Left Masseter	16.8 ± 3.2	10.2 ± 2.7	−39.3%	< 0.001	17.2 ± 3.3	8.5 ± 2.2	−50.6%	< 0.001

In the present study, 980 nm laser showed superior efficacy compared to 635 nm, likely due to its deeper tissue penetration, which allows more effective modulation of nociceptors within muscle trigger points and enhanced neuromodulation of pain pathways. Moreover, 980 nm may enhance mitochondrial activity and blood flow in deeper muscles, contributing to longer-lasting reductions in pain and improved sEMG activity.

Our study demonstrated a significant improvement in maximum mouth opening (MMO) measured by digital caliper, consistent with Nasr et al. (2024) [14] who reported increased MMO using a 980 nm diode laser with eight sessions over one month.However, Fathy et al. (2021) [10] found no significant difference in MMO after six months, which may be related to differences in wavelength, fluence, or treatment protocol. Such discrepancies may also arise from differences in measurement methods (subjective vs. objective) or patient characteristics. Current literature lacks consensus on the optimal frequency and number of LLLT sessions; some recommend eight sessions twice weekly (Kurt et al. 2020) [15] others six sessions twice weekly (Benedicenti et al., 2015; Herpich et al., 2020) [16, 17] and some suggest ten sessions (Madani et al., 2020) [18].

Mahmoud et al. (2024) [19] showed that increasing session numbers (up to three per week) using a 940 nm laser improved pain, MMO, and quality of life in patients with myofascial trigger points.

Not all studies precisely measure mandibular movements such as protrusive and lateral excursions, which limits understanding of PBMT’s effect on these functions. In this study, a significant improvement in lateral excursion was observed, consistent with Chami et al. (2020) [20] who reported an increase in lateral movement following treatment with an 808 nm diode laser over three sessions in one week. Similarly, Madani et al. (2020) [18] found significant improvements in both lateral and protrusive movements after LLLT with an 808 nm laser applied twice weekly for five weeks, with effects maintained at one-month follow-up.

Statistical analysis of EMG data revealed a significant decrease in muscle activity in both groups, with a more pronounced reduction in the 980 nm group. This aligns with clinical improvements in masticatory efficiency and reduced muscle tenderness, supporting the neuromodulatory effects of PBMT in TMD and myofascial pain syndrome (MPS). Surface electromyography performed during Maximum clenching showed that 980nm laser therapy effectively reduced sEMG activity in the masseter and temporalis muscles, indicating muscle relaxation and decreased hyperactivity. These findings are consistent with Leal de Godoy et al. (2017) [21] who reported significant sEMG reduction after 780 nm infrared laser therapy in TMD patients, and with Shousha et al. (2021) [22] who demonstrated similar effects using a 940 nm laser over ten sessions.

Both groups received PBMT twice weekly for ten sessions with a one-month follow-up. The 635 nm diode laser (0.5 W, CW, 60 s per trigger point) provided rapid analgesic effects within three days by targeting superficial tissues, enhancing mitochondrial activity, reducing inflammation, and promoting tissue repair. In contrast, the 980 nm diode laser (0.2 W, CW, 60 s on each trigger points) produced more sustained improvements, with significant enhancement in Mandibular function and greater reductions in EMG activity observed by day 7. Overall, while the 635 nm wavelength offered faster short-term pain relief, the 980 nm wavelength demonstrated superior long-term efficacy in reducing myofascial pain and improving masticatory function through deeper neuromodulatory effects, as confirmed by EMG analysis. Clinically, these results correspond to a meaningful improvement in MMO (7.6 mm for 635 nm and 10.2 mm for 980 nm) and VAS reduction (62% for 635 nm and 73% for 980 nm), highlighting the practical benefits for patients.

## Conclusions

This randomized controlled trial demonstrates that PBMT with 635 nm and 980 nm diode lasers alleviates pain, improves muscle activity, and restores Mandibular function in myofascial pain syndrome. These improvements translate into clinically meaningful gains in jaw function and pain relief, with 980nm showing superior long-term efficacy.

### Clinical implications

Clinicians may tailor PBMT wavelength selection based on patient needs: the 635 nm diode laser provides rapid short-term analgesia, whereas the 980 nm diode laser offers more sustained improvements in pain reduction and mandibular function. Incorporating PBMT into multidisciplinary management of MPS may enhance treatment outcomes and reduce dependence on pharmacological therapies.

### Limitations

This study is limited by a small sample size (*n* = 30), short follow-up duration (one month), single-center recruitment, and reliance on subjective pain measures. Additionally, the unequal fluence between the 635 nm and 980 nm groups (38.22 vs. 15.28 J/cm²) may have influenced treatment outcomes, and the absence of a placebo/sham control limits the ability to isolate specific PBMT effects.

### Future directions

Future studies should include larger, multi-center samples with longer follow-up, incorporate placebo/sham controls, and use objective assessment tools such as ultrasound to evaluate muscle changes. Further research should also investigate the cellular and molecular mechanisms underlying differential responses between wavelengths and evaluate additional functional outcomes, such as masticatory efficiency and repetitive muscle activity. Standardizing fluence and optimizing session protocols will help clarify PBMT outcomes.

## Supplementary Information

Supplementary Material 1

## Data Availability

The datasets used and/or analysed during the current study are available from the corresponding author on reasonable request.

## Abbreviations

PBMT: Photobiomodulation Therapy
LLLT: Low-Level Laser Therapy
TMD: Temporomandibular Disorders
MPS: Myofascial Pain Syndrome
sEMG: Surface electromyography
RCT: Randomized Controlled Trial
VAS: Visual Analogue Scale
