# Supplementary material for: Efficacy of photobiomodulation therapy using 980 nm versus 635 nm diode lasers for treatment of myofascial pain : a randomized controlled trial
Source: BMC Oral Health. 2025 Oct 2;25:1511. doi: 10.1186/s12903-025-06971-7 (PMC12490059; doi:10.1186/s12903-025-06971-7)
Supplement: Supplementary file 1 — Supplementary Material 1 [file 12903_2025_6971_MOESM1_ESM.pdf]

Figure 1: CONSORT 2025 Flow Diagram

Flow diagram of the progress through the phases of a randomised trial of two groups (that is, enrolment, intervention allocation, follow-up, and data analysis)

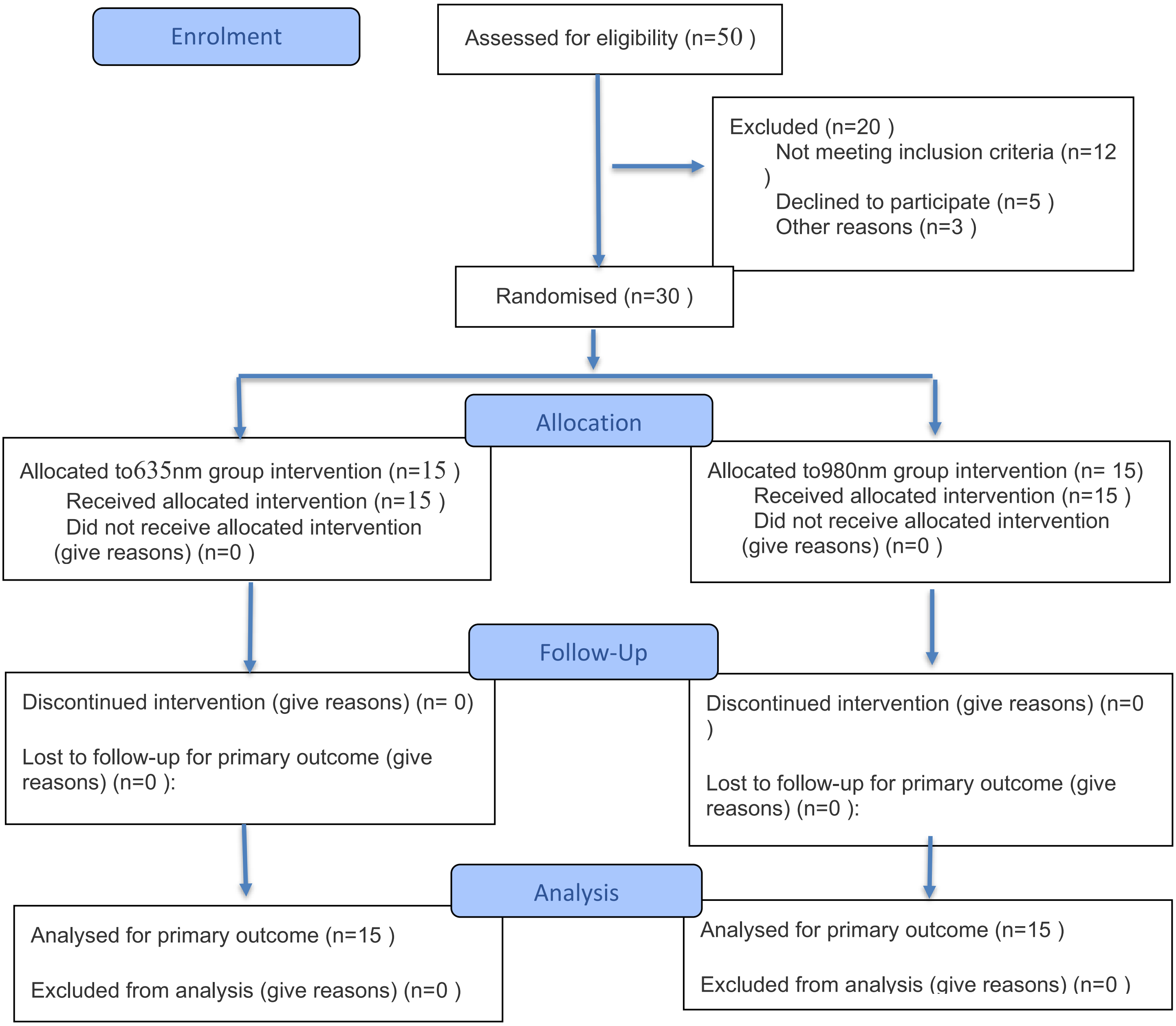

Citation: Hopewell S, Chan AW, Collins GS, Hróbjartsson A, Moher D, Schulz KF, et al. CONSORT 2025 Statement: updated guideline for reporting randomised trials. BMJ. 2025; 388:e081123. https://dx.doi.org/10.1136/bmj-2024-081123
© 2025 Hopewell et al. This is an Open Access article distributed under the terms of the Creative Commons Attribution License (https://creativecommons.org/licenses/by/4.0/), which permits unrestricted use, distribution, and reproduction in any medium, provided the original work is properly cited.
